# Prevalence of Skin Sensitization to Pollen of Date Palm in Marrakesh, Morocco

**DOI:** 10.1155/2017/6425869

**Published:** 2017-02-08

**Authors:** Hind Serhane, Lamyae Amro, Hafsa Sajiai, Abdelhaq Alaoui Yazidi

**Affiliations:** Department of Pulmonology-Allergology, Arrazy Hospital and Mohammed VI University Hospital, PCIM Laboratory, Cadi Ayyad University, Marrakesh, Morocco

## Abstract

*Background*. Date palm's pollen has been identified as a source of allergy; the rate of sensitization of this pollen is between 6 and 29%.* Objective*. To determine the prevalence of sensitization to date palm in Marrakesh and to identify the clinical profile.* Patients and Methods*. This study is based on a questionnaire and the prick test on 7 allergens, in population aged 5 years and above with clinical symptoms suggesting allergic diseases, from November 2012 to February 2013 in Marrakech.* Results*. We included 468 patients (women: 79.5%). The prick tests were considered interpretable in 467 cases. The prevalence of skin sensitization to pollen of date palm was 6.6%. The 31 cases of sensitization to date palm involved 7 men and 24 women with an average age of 37.5 years. Cutaneous monosensitization to date palm's pollen was observed in 2 cases. Asthma, rhinitis, and conjunctivitis were recorded, respectively, in 48.4%, 93.5%, and 67.7%.* Conclusion*. Skin sensitization to pollen of date palm does not seem unusual among allergic patients in Marrakech and is comparable to that found in Casablanca (7%), Barcelona (6.6%), and Cartagena (6.1%).

## 1. Introduction

Marrakesh palm groves are considered as a historical monument (since early last century, it is the only natural grouping of date palms, located at north of the Atlas Mountains) [1]. Date palm's pollen has been identified as a source of allergy, with sensitization rates among patients with respiratory allergies up to around 13% in the United Arab Emirates [2], 23% in Saudi Arabia [3], and from 6% to 29% in Spain [4, 5]. The lack of national and Maghreb data justified the realization of this study to determine the prevalence of sensitization in Marrakesh and to identify the clinical and allergic profile of sensitized patients to date palm.

## 2. Patients and Methods

This is a descriptive cross-sectional study based on a questionnaire and the realization of a prick test (PT) on 7 allergens provided by Stallergenes Laboratory whose references are 315P3-ST for* Dermatophagoides pteronyssinus* (DP), 314P3-ST for* Dermatophagoides farinae* (DF), 688P3-ST for pollen of the 5 grasses, 651P3 for olive tree pollen, 626P3 for cypress tree pollen, 668P3 for date palm pollen, and 301P3 for German cockroach. This study was conducted over four-month period from 1 November 2012 until the end of February 2013 at Marrakesh in six health centers, located near date palms implantation. We included in the study any patient aged 5 years and more, with clinical symptoms suggesting asthma and/or rhinitis and/or conjunctivitis and/or cutaneous manifestations such as eczema or urticaria. We used the histamine dihydrochloride (10 mg/ml) as a positive control to ensure skin reactivity and glycerol containing the diluent of the prick test solution as a negative control to eliminate dermographism. We considered a test positive if the wheal diameter obtained was 3 mm greater than the negative control and/or 50% greater than the diameter of the positive control. Data analysis was performed by the software Epi Info version 6, and statistical comparisons were made by the Chi-2 test.

## 3. Results

**Figure 1:**
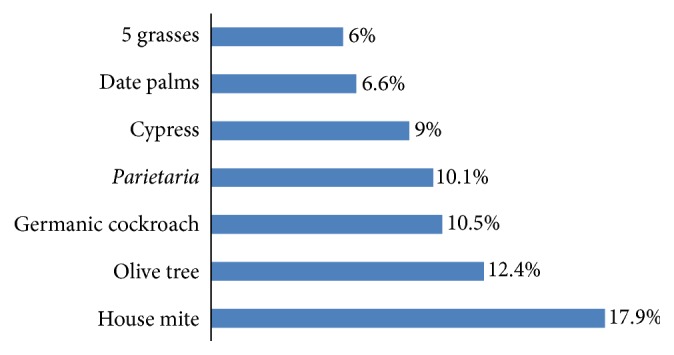
Prevalence of positive skin prick tests to allergens.

In our study, we included 468 patients, 372 women and 96 men with female predominance of 79.5%. The average age was 34.9 +/− 16.5 years with extremes ranging from 5 to 83 years. Asthma, rhinitis, and conjunctivitis were noted, respectively, in 53.4%, 73.7%, and 57.1%. The prick tests were performed in 468 cases and considered interpretable in 467 cases. They are considered positive in 190 cases or 40.7% of the total studied population. Allergy to pollen of date palm (6.6%) came in the sixth skin sensitization after house mite dust (17.9%), olive tree (12.4%), German cockroach (10.5%),* Parietaria* in (10.1%), and cypress tree (9%) and before the 5 grasses (6%). Skin sensitization to different allergens is grouped in Figure 1. Skin sensitization to pollen of date palm was found in 31 patients (6.6%). The average age of these patients was 37.5 years (range from 9 to 56 years). The maximum of patients sensitized to the date palm was found in the age group 30–49 years (16 patients) (*p* = 0.018). In fact there is no female predominance because 7.3% of women sensitized to pollen of date palm against 6.5% men (*p* = 0.8). 16 patients of the 31 (51.6%) sensitized to the date palm reported the presence of date palm tree in their immediate surroundings and only 3 patients were directly exposed to this tree. Cutaneous monosensitization to date palm was noted in only 2 cases, among 29 other patients; skin sensitization was most commonly associated with the cockroach sensitization in 13 cases/29 which corresponds to 44.8% of cases, with cypress sensitization in 11 cases/29 (37.9%), with the olive tree sensitization in 9 cases/29 (31.0%), with the* Parietaria* sensitization in 8 cases/29 (27.6%), with the 5-grass sensitization in 8 cases/29 (27.6%), and with the mite in 7 cases/29 (24.1%). In terms of the clinical history and the physical examination, allergic manifestations were diverse. But it appears that rhinitis was an almost constant symptom and it was found globally in 29 cases/31 (93.5%) (*p* = 0.01). Asthma was found globally in 15 cases/31 (48.4%) (*p* = 0.6). Conjunctivitis was found in 21 cases/31 (67.7%) (*p* = 0.3).  Table 1 includes the clinical profile of patients sensitized to pollen of date palm. The asthma symptoms were aggravated by exposure to pollen of date palm in 20% of cases (*p* = 0.04); this rate was at 23.3% for rhinitis (*p* = 0.02) and at 23.8% for conjunctivitis (*p* = 0.1). The average size of the wheal due to the date palm PT was 4.9 mm (range from 3 to 7 mm) against 7.6 mm +/− 2 mm for others allergens, in the general population studied.

## 4. Discussion

Different types of studies have focused on determining the prevalence of allergy to pollen of date palm, but the populations were heterogeneous and not well defined. Furthermore, the used allergen extracts were not standardized; we used various allergens and various concentrations. All these factors make it difficult to compare the results of all the work developed significantly. In Spain, five studies have investigated the prevalence of skin sensitization to pollen of date palm in patients with respiratory allergic symptoms or pollinosis. In these patients, the prevalence of skin sensitization was between 6.11% and 29.41% [4–8]. In Brussels, in 2006, Mahillon et al. conducted a study on 59 patients followed up for persistent rhinitis and exposed to indoor decorative plants in their own home. They found a prevalence of skin sensitization to palm tree at 10.16% [9]. In our study, the prevalence of skin sensitization to date palm in patients with rhinitis was 8.4%. In UAE, two studies in Al Ain city involving patients with respiratory allergic symptoms noted prevalence between 3.6% and 13.8% [2–10]. In Saudi Arabia, Almogren conducted a retrospective study between January 2003 and March 2004, on the analysis of skin testing of patients referred for allergic reactions to the allergy clinic at the King Khalid University Hospital, Riyadh. He found a prevalence of skin sensitization to pollen of date palm at 23% (15% in adults and 35% in children) [3]. In our study, the prevalence of skin sensitization to date palms was 7.1% and 4%, respectively, in adults and children under 15 years. In Vietnam To My and Raffard noted skin sensitization to date palms of 1.9% in 108 subjects with confirmed asthma living in Ho Chi Minh City [11]. In Algeria, a study by Gharnaout et al. on 82 patients consulting for respiratory allergic symptoms (rhinitis or bronchial asthma and/or conjunctivitis) had found that 16% of patients had skin sensitization to date palm [12]. In Morocco, a prospective study by Elkhattabi et al. in Casablanca found a prevalence of skin sensitization to pollen of date palm at 7% [13] which was close to that found in our study in Marrakech. Allergy to date palm can occur at any age. Indeed, the average age of patients sensitized to date palm is variable depending on the population studied (children or adults exclusively or mixed). In the study of Gharnaout et al. conducted in Biskra (Algeria), the average age in patients sensitized to date palm was 34.15 years [12]. Huertas et al. found no statistically significant age difference between patients sensitized and not sensitized to pollen of date palm with, respectively, 30.52 ± 18.45 years and 27 years ± 16.86 years [14]. In our study, we noted a female predominance (77.4%), while Gharnaout et al. noted a male predominance, respectively, in 69.23% and 54.2% of cases [12–14]. In all series, rhinitis was the most common clinical manifestation in patients sensitized to pollen of date palm. In fact, it was present in 93.5% of our patients, 89.02% of patients in the study of Gharnaout et al. [12], and 97.9% of patients in the study of Huertas et al. [14]. Conjunctivitis was the most common condition after rhinitis. Gharnaout et al.'s study established a similar observation with a rate of 63.41% [12]. In our study, asthma was the least common clinical manifestation in patients sensitized to pollen of date palm with a rate of 48.4%. A similar rate has been reported by Gharnaout et al. (48.78%) [12]. While Huertas et al.'s study found a rate of asthma at 72.9% in patients sensitized to date palm, it was significantly higher in patients sensitized to date palm than in nonsensitized patients (45.8%) [14]. Allergy to pollen of date palms is frequently associated with polysensitization [13]. Huertas et al. who were interested in allergic profile of patients sensitized to date palm had found that the distribution of atopic patients by number of pollen sensitization types varied according to the existence or not of the patients sensitized to date palm. 74.51% of patients with negative skin tests to date palms had under 4 skin sensitization types associated, whereas all patients sensitized to date palm had more than 4 skin sensitization types associated [14]. This could be explained by the fact that sensitization to pollen of date palm in the Mediterranean is an expression of a pan allergen sensitization which is probably a profilin [8]. The same author in another study found that the group of patients sensitized to pollen of date palm had a high sensitization rate to different pollens compared to nonsensitized patients (7.25 against 3.31) and this could be a severity indicator of pollinosis in the region, because these polysensitized patients had high risk of bronchial asthma (72.9% against 45.8%) [14].

## 5. Conclusion

Allergy to pollen of date palm does not seem to be rare around the Mediterranean basin. Patients sensitized to pollen constitute a homogeneous group of patients characterized by frequent polysensitization and a clinical feature dominated by allergic rhinitis which is the most common clinical manifestation. Therefore, we recommend including the date palm allergen among the routine allergic skin prick tests.

## Figures and Tables

**Table 1:** Clinical manifestations of patients sensitized to pollen of date palms.

Clinical manifestations	Number of patients	%
Asthma alone	1	3,2
Rhinitis alone	4	12,2
Conjunctivitis alone	1	3,2
Asthma and rhinitis	5	16,1
Asthma and conjunctivitis	0	0
Asthma and rhinitis and conjunctivitis	9	29
Rhinitis and conjunctivitis	11	35,4
